# Joint Transmit Power Allocation and Splitting for SWIPT Aided OFDM-IDMA in Wireless Sensor Networks

**DOI:** 10.3390/s17071566

**Published:** 2017-07-04

**Authors:** Shanshan Li, Xiaotian Zhou, Cheng-Xiang Wang, Dongfeng Yuan, Wensheng Zhang

**Affiliations:** 1 Shandong Provincial Key Laboratory of Wireless Communication Technologies, School of Information Science and Engineering, Shandong University, Jinan 250100, China; 201511874@mail.sdu.edu.cn (S.L.); dfyuan@sdu.edu.cn (D.Y.); zhangwsh@sdu.edu.cn (W.Z.); 2 Institute of Sensors, Signals and Systems, School of Engineering and Physical Sciences, Heriot-Watt University, Edinburgh EH14 4AS, UK; cheng-xiang.wang@hw.ac.uk

**Keywords:** Simultaneous Wireless Information and Power Transfer (SWIPT), Orthogonal Frequency Division Multiplexing-Interleave Division Multiple Access (OFDM-IDMA), iterative Multi-User Detection (iterative MUD), Signal to Interference and Noise Ratio (SINR), Power Allocation

## Abstract

In this paper, we propose to combine Orthogonal Frequency Division Multiplexing-Interleave Division Multiple Access (OFDM-IDMA) with Simultaneous Wireless Information and Power Transfer (SWIPT), resulting in SWIPT aided OFDM-IDMA scheme for power-limited sensor networks. In the proposed system, the Receive Node (RN) applies Power Splitting (PS) to coordinate the Energy Harvesting (EH) and Information Decoding (ID) process, where the harvested energy is utilized to guarantee the iterative Multi-User Detection (MUD) of IDMA to work under sufficient number of iterations. Our objective is to minimize the total transmit power of Source Node (SN), while satisfying the requirements of both minimum harvested energy and Bit Error Rate (BER) performance from individual receive nodes. We formulate such a problem as a joint power allocation and splitting one, where the iteration number of MUD is also taken into consideration as the key parameter to affect both EH and ID constraints. To solve it, a sub-optimal algorithm is proposed to determine the power profile, PS ratio and iteration number of MUD in an iterative manner. Simulation results verify that the proposed algorithm can provide significant performance improvement.

## 1. Introduction

Allowing multiple users to communicate with each other in the same network, Multiple Access (MA) has been considered as one of the most important techniques in the area of wireless communications. It is widely recognized that the existing MA schemes can be classified into two categories, namely, Orthogonal Multiple Access (OMA), and Non-orthogonal Multiple Access (NOMA) [1]. In the former case, different users access to the network utilizing different resource blocks (i.e., frequency, time, or orthogonal codes). The Multiple Access Interference (MAI) is therefore absent, leading to the lower detection complexity. While for the latter, multiple users are served with the same resource block, where MAI exists but can be mitigated through Successive Interference Cancellation (SIC). According to information theory, NOMA is superior than OMA in terms of Spectral Efficiency (SE). As a consequence, despite of the higher complexity paid for interference cancellation, NOMA is still expected to be the most potential candidate for the future communication networks.

Among numerous NOMA schemes, Interleave Division Multiple Access (IDMA) was proposed in [2] and has drawn increasing research interests. Different from the traditional NOMA operated in power domain, in IDMA signals from different users are labelled by different interleaving patterns. The iterative Multi-User Detection (MUD) is utilized to recover the signals of each user at the receiver. It is reported that the signal-user performance can be achieved by IDMA with a substantial number of users, given MUD the sufficient number of iterations together with proper transmit power allocation [2]. In addition, IDMA can be easily integrated with other existing MA paradigms, such as Orthogonal Frequency Division Multiplexing (OFDM) [3]. The resulted OFDM-IDMA system inherits the advantages of both OFDM and IDMA, and hence is considered as one promising MA approach for the fifth generation (5G) wireless communications. Despite of the excellent performance, the implementation of OFDM-IDMA is still a difficult task due to the employment of iterative MUD. In fact, the attractive features of OFDM-IDMA, such as the high spectral efficiency and good error-rate performance, rely on the iterative MUD employed at the receiver side. However, such iterative detection is more computation costly than traditional non-iterative one, leading to higher power consumption. In some power-constrained scenarios of 5G such as Internet of Things (IoT) and Wireless Sensor Networks (WSN), the receive nodes (RNs) are usually supplied by batteries with limited power [4,5]. Consequently it is difficult to implement OFDM-IDMA into such case since the RNs are short of enough energy to execute MUD with sufficient iteration numbers.

On the other hand, Simultaneous Wireless Information and Power Transfer (SWIPT) is a novelly proposed concept who is expected to alleviate such energy-constrained problem. Based on the fact that the Radio Frequency (RF) signals carry both information and energy, in SWIPT RNs are allowed to harvest the energy from the received signals while decoding them [6]. By properly coordinating the wireless information and power propagation through Power Splitting (PS), Time Switching (TS) or Antenna Switching (AS), the wireless recharging can be realized without interrupting the data transmission [7,8].

The early literatures about SWIPT in multi-user scenario mainly focused on the OMA schemes, i.e., OFDM in [9,10,11] and Time Division Multiple Access (TDMA) in [12]. While the research on the implementation of SWIPT in the power domain NOMA system is conducted in [13,14,15]. In [13], the authors proposed a wireless-powered NOMA system, where the RNs are wireless recharged by Source Receive (SN) during the downlink transmission and then use the harvested energy to transmit their own information to SN during uplink phase. A joint design on the energy harvest time-slot and decoding orders was also proposed to balance the maximum achievable data rate and fairness among the RNs. [14] adopted a similar system to that in [13] but focused on the joint optimization of the transmit power of SN along with the tradeoff between energy harvest and information transmission durations. [15] considered the cooperative multi-user networks based on the SWIPT-NOMA scheme. And the main focus was on the relay-user selection strategies, with the purpose of achieving a lower outage probability or better throughput.

Inspired by the previous work mentioned above, in this paper we propose the SWIPT aided OFDM-IDMA scheme. Our purpose is to provide enough power to RNs through wireless energy harvesting, so that the iterative MUD is capable to work with sufficient number of iterations. Such purpose along with the unique properties of OFDM-IDMA, make the proposed system to be different from the previous work in two aspects:

- From the aspect of EH, in the proposed system the harvested energy is fully devoted to recharging the circuits of MUD. As a consequence, the energy harvesting requirement is related to the iteration number of MUD, which should be treated as variables rather than a fixed constant as that in traditional detectors.
- From the aspect of Information Decoding (ID), the performance of the proposed system is determined by the final Signal to Interference plus Noise Ratio (SINR) after iterative decoding. Hence it relies on the iteration number of MUD, the number of RNs and also the initial power assigned to them. Such iterative detection process is difficult to track, which makes the system design to be more complicated.

Bearing these unique features in mind, in this paper we focus on the transmit power minimization problem in the proposed system with two constraints, which are (1) providing sufficient energy for MUD to work; and (2) satisfying the desired Bit Error Rate (BER) requirements of the RNs. Against such objective, we first setup the constraint for EH, which is a non-linear function with respect to the transmit power and is also sensitive to the iteration number of MUD. Then we investigate the explicit relationship between the final SINR and BER performance of SN, where the iterative MUD is taken into account with the help of SNR evolution function [16]. The power minimization problem is therefore formulated, which subjects to not only the transmit power assignment and power splitting ratio, but also the iterations number of MUD for each RN. As the optimal solution to the original problem is hardly obtained, we decompose the original problem into two independent sub-problems, where one solely considers the power allocation with the assumption of given PS ratio and number of iterations, and the other one deals with the optimization of PS ratio and iteration number with fixed power profile. We then propose a sub-optimal algorithm by solving these two sub-problems in an iterative manner. And the solution to the original solution can be finally approached through such algorithm.

Specifically, the main contributions of this work are summarized as follows:

- We introduce the concept of Simultaneous Wireless Information and Power Transfer (SWIPT) into OFDM-IDMA, resulting in the proposed SWIPT-aided OFDM-IDMA system. Not only inherits the advantages of OFDM-IDMA, the proposed system also allows the SN to wireless recharge the RNs along with information transmission. The energy limitation of RNs is therefore alleviated and the iterative MUD can be carried out with sufficient iteration numbers. Hence the proposed system is suitable for the power-constraint scenarios of 5G, such as Internet of Things and Wireless Sensor Networks.
- With the proposed system, we also investigate the impacts of iteration number of MUD, BER requirements, as well as PS ratio on the transmit power of SN. Aiming at minimizing the transmit power of SN, we propose a sub-optimal algorithm to jointly coordinate the power allocation and power splitting in an iterative approach. The energy efficiency of the whole system is significantly improved through our algorithm.

The rest of this paper is organized as follows. In Section 2, we describe the proposed SWIPT-aided OFDM-IDMA model. The analysis on the iterative MUD, as well as the problem formulation are also included in this section. We then propose the sub-optimal algorithm in Section 3, along with the discussion related to the impacts of the three readily optimized parameters on the system performance. Simulations are provided in Section 4 to evaluate the performance of the proposed algorithm. And the concluding remarks and future works are drawn in Section 5.

## 2. System Model and Problem Formulation

### 2.1. System Model

We consider a wireless access network with one SN transmitting to *K* RNs. The SN who has constant power supplies, can act as both the information transmitter and power beacon. While the RNs are battery-powered who expect to receive the information from SN and also be wirelessly recharged by SN. The channel between SN and each RN is assumed to experience both large scale fading and frequency-selective multi-path Rayleigh-fading. The number of fading paths equals to *L*. To combat such frequency selective fading, Orthogonal Frequency Division Multiplexing (OFDM) is employed. At the SN, the data for each RN is first encoded by the same encoder but permuted by different chip-level interleavers. The resulted sequences are then linearly superimposed together and modulated onto the subcarriers readily for transmission. At the *k*-th RN, the received signal on the *n*-th subcarrier is:

(1) $$\begin{array}{cc} Y_{k}\left(n\right) & =H_{k}\left(n\right)\sum_{i=1}^{K}\sqrt{P_{i}}X_{i}\left(n\right)+\omega \\ & =H_{k}\left(n\right)\sqrt{P_{k}}X_{k}\left(n\right)+\zeta_{k}\left(n\right)\;,\;\;n=1,\cdots ,N \end{array}$$

where $H_{k}\left(n\right)$ contains not only the frequency channel response of the *k*-th RN on subcarrier *n*, but also the path-loss from SN to RN *k*. $X_{k}\left(n\right)$ is the transmitted symbol for the *k*-th RN on subcarrier *n*. $P_{k}$ is the transmit power per-subcarrier assigned to the *k*-th RN. *N* is the number of subcarriers in one OFDM block. ω denotes the Additive White Gaussian Noise (AWGN) follows $N∼(0,\sigma^{2})$. $\zeta_{k}\left(n\right)$ represents the interference plus noise part with respect to $X_{k}\left(n\right)$, which can be further expressed as:

(2) $$\begin{array}{c} \zeta_{k}\left(n\right)=H_{k}\left(n\right)\sum_{k^{\prime }\neq k}^{K}\sqrt{P_{k^{\prime }}}X_{k^{\prime }}\left(n\right)+\omega \;,\;\;n=1,\cdots ,N\;. \end{array}$$

**Figure 1:**
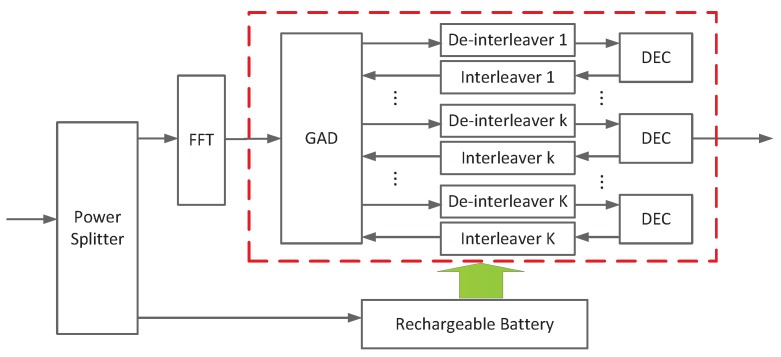
The receiver structure of the *k*-th RN.

It is assumed that the proposed SWIPT aided system works under the PS mode, as shown in Figure 1. Then the received signal at each RN is partitioned into two parts through a power splitter. To be specific, an $\rho_{k}$ portion of the signal power is fed to the iterative MUD for ID while the remaining signal power is gathered by the Energy Receiver (ER), where $0\leq \rho_{k}\leq 1,\forall k$. The average received RF power at the ER of each SN during one OFDM block transmission is expressed as:

(3) $$\begin{array}{c} P_{k}^{EH}=\left(1-\rho_{k}\right){\left|H_{k}\right|}^{2}\sum_{k=1}^{K}P_{k} \end{array}$$

where $|H_{k}{|}^{2}=\sum_{n=1}^{N}{\left|H_{k}\left(n\right)\right|}^{2}$ is the accumulated channel gain of RN *k* across the entire OFDM block [17].

Note that the harvested RF power has to be converted to the direct current (DC) electricity, in order to be stored and utilized by RNs. It has been proved by field experiments that such energy conversion is a non-liner process, where the conversion efficiency would increase at first and then decrease as the input RF power rises [18,19]. To describe such behavior, we adopt the non-linear EH model proposed in [20,21]. Based on that, the actually received DC energy of *k*-th RN can be expressed as:

(4) $$\begin{array}{c} \Phi_{k}=\frac{\left(1+e^{a_{k}b_{k}}\right)\Psi_{k}-M_{k}^{2}}{1+e^{a_{k}b_{k}}-M_{k}} \end{array}$$

where

(5) $$\begin{array}{c} \Psi_{k}=\frac{M_{k}}{1+e^{-a_{k}\left(P_{k}^{EH}-b_{k}\right)}} \end{array}$$

is the logistic function with respect to the received RF power $P_{k}^{EH}$. $M_{k}$, $a_{k}$ and $b_{k}$ are the fixed parameters determined by the detailed EH circuit specifications.

On the other hand for information decoding, the initial received SINR for the *k*-th RN at subcarrier *n* can be expressed as:

(6) $$\begin{array}{c} \lambda_{k}^{0}\left(n\right)=\frac{\rho_{k}{\left|H_{k}\left(n\right)\right|}^{2}P_{k}}{\rho_{k}{\left|H_{k}\left(n\right)\right|}^{2}\sum_{k^{\prime }\neq k}^{K}P_{k^{\prime }}+\sigma^{2}} \end{array}$$

where the bandwidth of subcarrier is assumed to be unity for the ease of analysis.

### 2.2. Iterative Multi-User Detection and SNR Evolution

Recall that an iterative MUD is employed at each RN of the proposed system, which is comprised of an Gaussian Approximation Detector (GAD) and K signal-user *a posteriori probability* decoders (APP DECs) working in an iterative manner.

To decode the desired signal, say $X_{k}\left(n\right)$ for RN *k*, the GAD first treats $\zeta_{k}\left(n\right)$ in (2) as the Gaussian random variable based on Central Limit Theorem. The extrinsic information about $X_{k}\left(n\right)$; defined as $L_{e}\left(X_{k}\left(n\right)\right)$, can be generated following an soft-input soft-output (SISO) estimation procedure given the observation of $Y_{k}\left(n\right)$ and the *a priori* information provided by APP DECs [22]. Then $L_{e}\left(X_{k}\left(n\right)\right)$s are de-interleaved and fed to the APP DECs as the *a priori* information. According to the standard APP decoding rule [23], the DEC outputs the corresponding extrinsic information and sends back its interleaved version $L_{c}\left(X_{k}\left(n\right)\right)$ to the GAD as the *a priori* information to update the estimate of $\zeta_{k}\left(n\right)$. The MAI will be mitigated gradually with such extrinsic information exchanging in an iterative manner. After the *q*-th iteration, the residual interference with respect to the *k*-th RN on subcarrier *n* can be denoted as [24]:

(7) $$\begin{array}{c} V^{q}\left\{\zeta_{k}\left(n\right)\right\}=\rho_{k}{\left|H_{k}\left(n\right)\right|}^{2}\sum_{k^{\prime }\neq k}^{K}P_{k^{\prime }}V^{q}\left\{X_{k^{\prime }}\left(n\right)\right\}+\sigma^{2} \end{array}$$

where V{·} denotes the variance of the corresponding variables. Note that $V^{q}\left(X_{k^{\prime }}\left(n\right)\right)$ represents the residual interference contributed by RN $k^{\prime }$ in current iteration, which can be calculated based on $L_{c}^{\left(q-1\right)}\left(X_{k^{\prime }}\left(n\right)\right)$ in the previous iteration as:

(8) $$\begin{array}{c} V^{q}\left\{X_{k^{\prime }}\left(n\right)\right\}=1-\tanh^{2}\left(\frac{L_{c}^{\left(q-1\right)}\left(X_{k^{\prime }}\left(n\right)\right)}{2}\right)\;. \end{array}$$

As a consequence, the final SINR with respect to $X_{k}\left(n\right)$ after the *Q*-th iteration can be obtained as:

(9) $$\begin{array}{c} \lambda_{k}^{Q}\left(n\right)=\frac{\rho_{k}{\left|H_{k}\left(n\right)\right|}^{2}P_{k}}{\rho_{k}{\left|H_{k}\left(n\right)\right|}^{2}\sum_{k^{\prime }\neq k}^{K}P_{k^{\prime }}V^{Q}\left\{X_{k^{\prime }}\left(n\right)\right\}+\sigma^{2}}\;. \end{array}$$

We can find that to get the final SINR of one specific RN *k*, it should take into account the variance value of other RNs on each subcarrier per-iteration. Apparently, one can utilize brute-force simulations to get (9). However, such an approach is time consuming and impractical to be employed for performance optimization. In fact, the performance of OFDM-IDMA is related to the *averaged SINR* per-subcarrier, thanks to the low-rate coding as well as the chip-level random permutation over the entire OFDM block. Based on that idea, an SNR evolution method was proposed in [2], where the SINR as well as the variance value of RN *k* per each subcarrier are approximated by their expectations over the entire OFDM block as:

(10) $$\begin{array}{c} \Lambda_{k}^{q}=E\left[\lambda_{k}^{q}\left(n\right)\right]\;,\;\;\;\;\;{\bar{V}}_{k}^{q}=E\left[V_{k}^{q}\left(n\right)\right]\;. \end{array}$$

The recursive relationship between them during each iteration can be characterized by the defined f(·) function as ${\bar{V}}_{k}^{q}=f\left(\Lambda_{k}^{q-1}\right)$, where f(·) is obtained by simulating the single RN system under the same channel conditions [2]. By substituting (9) and f(·) function into (10), we can get the averaged SINR updating rule as:

(11) $$\begin{array}{c} \Lambda_{k}^{q}=\frac{\rho_{k}{\left|H_{k}\right|}^{2}P_{k}}{\rho_{k}{\left|H_{k}\right|}^{2}\sum_{k^{\prime }\neq k}^{K}P_{k^{\prime }}f\left(\Lambda_{k^{\prime }}^{q-1}\right)+N\sigma^{2}}\;. \end{array}$$

It can be observed in (11) that the SINR updating of RN *k* is now related to the SINR rather than the variance values of its counterparts. It helps to facilitate the performance analysis of iterative decoding in OFDM-IDMA, with the easily obtained f(·) function. Following the same idea, the BER performance of RN *k* can also be semi-analytically tracked as ${\mathrm{BER}}_{k}=g\left(\Lambda_{k}^{Q}\right)$, where g(·) is also obtained through simulating the single RN system similar to that of f(·). Details about the SNR evolution method c.f. [2,24].

### 2.3. Problem Formulation

In this subsection, we formulate the optimization problem of the proposed SWIPT aided OFDM-IDMA system. Recall that our purpose is to minimize the transmit power of SN, in the condition that both EH and ID requirements of RNs are satisfied. We will first derive these two constraints before formulating the power minimization problem.

- The Energy Harvesting (EH) Constraint
  In the proposed system, the harvest energy is utilized to recharge the circuits of MUD, so that the iterative decoding can work under sufficient number of iterations. To achieve that, the harvested DC energy should be no less than the one needed for iterative MUD, which can be expressed as:
  (12) $$\begin{array}{c} \Phi_{k}\geq E_{c}Q_{k}\; \end{array}$$
  where $E_{c}$ is the minimum required energy for MUD to work per iteration and $Q_{k}$ is the iteration number of the *k*-th RN. So far the EH constraint can be expressed as (12). Nevertheless, note that the objective is to minimize the transmit power of RF signals from SN. Hence when formulating the EH requirement, the direct and explicit relationship between $E_{c}Q_{k}$ and $P_{SN}$ is preferred, rather than that between $E_{c}Q_{k}$ and $\Phi_{k}$. Hence we further take (3)–(5) into (12) to substitute $\Phi_{k}$ with $P_{SN}$, and the resulted inequation can be expressed as:
  (13) $$\begin{array}{c} \left(1-\rho_{k}\right){\left|H_{k}\right|}^{2}P_{SN}\geq b_{k}-\frac{1}{a_{k}}\ln\left(\frac{M_{k}\left(1+e^{a_{k}b_{k}}\right)}{E_{c}Q_{k}\left(1+e^{a_{k}b_{k}}-M_{k}\right)+M_{k}^{2}}-1\right)\;,\;\;\;\forall k\in 1,\cdots ,K \end{array}$$
- The Information Decoding (ID) Constraint
  ID constraint guarantees the BER performance of the *k*-th RN, where the BER of RN *k* is expected to be lower than a given target. Recall that in Section 2.2, the g(·) function is introduced to depict the relationship between the BER performance and achievable SINR. Assuming the BER target of the *k*-th RN is $BER_{k}^{t}$, the corresponding SINR threshold to reach that can be obtained as $\Gamma_{k}\;=\;g^{-1}\left(BER_{k}^{t}\right)$. According to [2,16], g(·) is the monotonically decreasing function. As a consequence, the final SINR value of RN *k* after iterative decoding should be greater than $\Gamma_{k}$, so that to guarantee the desired BER performance. Based on (11), the ID constraint can be represented as:
  (14) $$\begin{array}{c} \Lambda_{k}^{Q_{k}}=\frac{\rho_{k}{\left|H_{k}\right|}^{2}P_{k}}{\rho_{k}{\left|H_{k}\right|}^{2}\sum_{k^{\prime }\neq k}^{K}P_{k^{\prime }}f\left(\Lambda_{k^{\prime }}^{Q_{k^{\prime }}-1}\right)+N\sigma^{2}}\geq \Gamma_{k}\;,\;\;\;\;\;\forall k\in 1,\cdots ,K \end{array}$$

With the preliminaries provided above, the optimization problem can then be formulated as follows:

**Original Problem Formulation (**OP**)**: *Given the minimum harvested energy constraints along with the Quality of Service (QoS) requirements of each RN, our objective is to minimize the total transmit power of SN, by jointly determining the **power allocation, power splitting and numbers of iterations** among all RNs. Such optimization problem can be summarized as follows:*

(15) $$\begin{array}{cc} \mathrm{OP}:\;\;\;\;\;\; & \;\;\;\min_{\begin{array}{c} \left\{\rho_{k}\right\},\left\{P_{k}\right\},\left\{Q_{k}\right\} \end{array}}\;\;\;\;\;\;P_{SN}=\sum_{k=1}^{K}\;\;\;\;P_{k}\\ & s.t.\;\;\;\;(13)\;,\;\;\;(14) \end{array}$$

where the first constraint guarantees the EH requirements and the second constraint denotes the ID requirements of RNs. It can be observed that the iterative decoding process is taken into account in (14), where the final SINR value of each RN is sensitive to not only the initial power assignment and PS ratio, but also to their number of iterations $\left\{Q_{k}\right\}$, as well as the achievable SINR in previous iteration. In addition, $\left\{Q_{k}\right\}$ also affects the EH requirements in (13). As a result, to get a promising solution to OP, $\left\{Q_{k}\right\}$ should be optimized along with $\left\{P_{k}\right\}$ and $\left\{\rho_{k}\right\}$.

## 3. The proposed Power Allocation and Splitting Algorithm

We in this section, propose the sub-optimal algorithm to solve the optimization problem concluded in OP. Since OP is untrackable and hard to solve, we propose to obtain $\left\{P_{k}\right\}$, $\left\{Q_{k}\right\}$ and $\left\{\rho_{k}\right\}$ in an iterative manner. To be specific, we first optimize $\left\{P_{k}\right\}$ with fixed $\left\{Q_{k}\right\}$ and $\left\{\rho_{k}\right\}$, and then update $\left\{Q_{k}\right\}$ and $\left\{\rho_{k}\right\}$ with the optimized $\left\{P_{k}\right\}$. The obtained results can then in turns help to update $\left\{P_{k}\right\}$ again iteratively.

### 3.1. Optimization of $\left\{P_{k}\right\}$ with Fixed $\left\{\rho_{k}\right\}$ and $\left\{Q_{k}\right\}$

We first optimize $\left\{P_{k}\right\}$ with pre-determined $\left\{\rho_{k}\right\}$ and $\left\{Q_{k}\right\}$. As stated in OP, the optimal solution to OP should satisfy both the EH constraint (13) and ID one (14). However, with the assumption that $\left\{\rho_{k}\right\}$ and $\left\{Q_{k}\right\}$ are already known, we can decouple these two constraints with each other and denote $P_{\mathrm{EH}}^{*}$ and $P_{\mathrm{ID}}^{*}$ are the minimum transmit power by solely considering the EH and ID requirements, respectively. Then the feasible solution to OP can be approximated as:

(16) $$\begin{array}{c} P_{\mathrm{SN}}^{*}\approx \max\left\{P_{\mathrm{EH}}^{*}\;,P_{\mathrm{ID}}^{*}\right\}\;. \end{array}$$

Note that (16) can hold only if $\left\{\rho_{k}\right\}$ and $\left\{Q_{k}\right\}$ are already fixed. Apparently, the minimum transmit power of SN is bounded by either EH or ID function at RN who requires more energy to achieve the target performance. We will leave the discussion on the relationship between $P_{\mathrm{EH}}^{*}$ and $P_{\mathrm{ID}}^{*}$ as well as their impact on the final solution at the end of this subsection, and focus on the approach to achieve them two here.

According to (13), $P_{\mathrm{EH}}^{*}$ is bounded by the RN who owns worst channel condition and largest iteration numbers, and can be easily expressed as:

(17) $$\begin{array}{c} P_{\mathrm{EH}}^{*}=\max_{\begin{array}{c} \left\{k=1,\cdots K\right\} \end{array}}\left\{\frac{\Pi_{k}}{\left(1-\rho_{k}\right){\left|H_{k}\right|}^{2}N}\right\} \end{array}$$

where

(18) $$\begin{array}{c} \Pi_{k}=b_{k}-\frac{1}{a_{k}}ln\left(\frac{M_{k}\left(1+e^{a_{k}b_{k}}\right)}{E_{c}Q_{k}\left(1+e^{a_{k}b_{k}}-M_{k}\right)+M_{k}^{2}}-1\right). \end{array}$$

We then focus on optimizing $P_{\mathrm{ID}}^{*}$ in the following. Note that the initial $\left\{Q_{k}\right\}$ should be set large enough to make the iterative MUD converge (To guarantee the convergence of MUD, $\left\{Q_{k}\right\}$ might be necessarily set over-large at initial stage. However, they will be optimized in the following steps.), where additional iterations would not bring extra benefits on cancelling the interference, i.e.,

(19) $$\begin{array}{c} f\left(\Lambda_{k}^{Q_{k}}\right)\approx f\left(\Lambda_{k}^{Q_{k}-1}\right)\;, \end{array}$$

In such a case, we can approximate $\Lambda_{k}^{Q_{k}}$ with $\Lambda_{k}$, where

(20) $$\begin{array}{c} \Lambda_{k}=\lim_{Q_{k}\to \infty }\Lambda_{k}^{Q_{k}}\;,\forall k \end{array}$$

is the theoretical upper bound of the achieved SINR when iteration number goes to infinity. Then, we can rewrite the first constraint in OP with (20) and get the sub-problem that optimizes $\left\{P_{k}\right\}$ solely with ID constraint as:

(21) $$\begin{array}{c} \;\;\;\min_{\left\{P_{k}\right\}}\;\;\;\;\;\;P_{\mathrm{ID}}=\sum_{k=1}^{K}\;\;\;\;P_{k} \end{array}$$

(22) $$\begin{array}{c} s.t.\;\;\;\;\;\;\;\Lambda_{k}=\frac{\rho_{k}{\left|H_{k}\right|}^{2}P_{k}}{\rho_{k}{\left|H_{k}\right|}^{2}\sum_{k^{\prime }\neq k}^{K}P_{k^{\prime }}f\left(\Lambda_{k^{\prime }}\right)+N\sigma^{2}}\geq \Gamma_{k} \end{array}$$

It can be found that the constraint (22) is relaxed without involving $\left\{Q_{k}\right\}$ and iterative decoding, which greatly facilitates our analysis. Intuitively speaking, to minimize the transmit power, the best option is to let the SINR of each RN to be just equal to its threshold as $\Lambda_{k}=\Gamma_{k}$. Unfortunately such favorable solution is almost inevitable to achieve. The reason falls in the fact that the achievable SINR of each RN is related not only to the initial power assignment $\left\{P_{k}\right\}$, but also to the SINR of other RNs, in the form of $P_{k^{\prime }}f\left(\Lambda_{k^{\prime }}\right)$. In other words, the SINR of RNs are associated with each other, and it is difficult to get the solution to the problem, by solely optimize $\left\{P_{k}\right\}$ with the only constraint (22). To overcome this, a new set of variables $\left\{\gamma_{k}\right\}$ is introduced to further decompose (22) into the following two constraints [25]:

(23) $$\begin{array}{c} \frac{\rho_{k}{\left|H_{k}\right|}^{2}P_{k}}{\rho_{k}{\left|H_{k}\right|}^{2}\sum_{k^{\prime }\neq k}^{K}P_{k^{\prime }}f\left(\Lambda_{k^{\prime }}\right)+N\sigma^{2}}\geq \gamma_{k} \end{array}$$

(24) $$\begin{array}{c} \Gamma_{k}\leq \gamma_{k}\leq \infty \;,\;\;\;\;\;\forall k\in 1,\cdots ,K \end{array}$$

where the strict constraint on $\left\{\Gamma_{k}\right\}$ is now relaxed with the new introduced variables $\left\{\gamma_{k}\right\}$. The promising solution to problem (21) could be obtained by minimizing both $\left\{\Gamma_{k}\right\}$ and $\left\{P_{k}\right\}$ jointly. Consequently, the sub-problem is re-formulated as follows [16]:

**Sub-Problem Formulation (**SP**)**: *Given the fixed iteration number, PS ratio, as well as the minimum SINR requirements* $\Gamma_{k}$ *with respect to certain BER performance of each RN, the objective of minimizing the total transmit power of SN can be achieved by jointly considering the **actually achievable SINR*** $\left\{\gamma_{k}\right\}$ *and **power allocation*** $\left\{P_{k}\right\}$ *among all RNs. The minimum transmit power, obtained through power allocation, should guarantee the actually achievable SINR of each RN to be also minimized but greater than* $\Gamma_{k}$. *Such a problem can be formulated as a cascaded one as:*

(25) $$\begin{array}{c} \begin{array}{cc} \mathrm{SP}:\;\;\; & \;\;\;\min_{\begin{array}{c} \left\{\gamma_{k}\right\} \end{array}}\left\{\begin{array}{c} \min_{\begin{array}{c} \left\{P_{k}\right\} \end{array}}\sum_{k=1}^{K}\;\;\;\;P_{k}\\ \;\;s.t.\;\frac{\rho_{k}{\left|H_{k}\right|}^{2}P_{k}}{\rho_{k}{\left|H_{k}\right|}^{2}\sum_{k^{\prime }\neq k}^{K}P_{k^{\prime }}f\left(\Lambda_{k^{\prime }}\right)+N\sigma^{2}}\geq \gamma_{k}\;, \end{array}\right\}\\ & s.t.\;\;\Gamma_{k}\leq \gamma_{k}\leq \infty \;,\;\;\;\;\;\forall k\in 1,\cdots ,K \end{array} \end{array}$$

where $\left\{\gamma_{k}\right\}$ is also involved into the optimization process together with $\left\{P_{k}\right\}$. So far SP is still a non-convex problem. But fortunately a similar problem for IDMA in uplink transmission has been successfully tackled with in [25], according to which SP can also be converted to a convex one by proper mathematical derivation.

To do so we first give the expression of optimal power allocation according to (23) as

(26) $$\begin{array}{c} P_{k}^{*}=\frac{\sigma^{2}\gamma_{k}^{*}}{\left(1-\sum_{k=1}^{K}\alpha_{k}\right)\left(1+\tilde{f}\left(\gamma_{k}^{*}\right)\right){\left|H\right|}_{k}^{2}\rho_{k}} \end{array}$$

where $\left\{\gamma_{k}^{*}\right\}$ is one feasible SINR solution to the outer problem illustrated in (25) and

(27) $$\begin{array}{c} \tilde{f}\left(\gamma_{k}^{*}\right)=\Lambda_{k}^{*}f\left(\gamma_{k}^{*}\right) \end{array}$$

(28) $$\begin{array}{c} \alpha_{k}=\frac{\tilde{f}(\gamma_{k}^{*})}{1+\tilde{f}(\gamma_{k}^{*})}\;. \end{array}$$

Based on (27) and (28), two new functions can be defined as

(29) $$\begin{array}{c} F\left(\alpha_{k}\right)=\left(1-\alpha_{k}\right){\tilde{f}}^{-1}\left(\frac{\alpha_{k}}{1-\alpha_{k}}\right) \end{array}$$

(30) $$\begin{array}{c} G\left(\alpha \right)=1-\sum_{k=1}^{K}\alpha_{k} \end{array}$$

where $\alpha =[\alpha_{1},\cdots ,\alpha_{K}]$ is the vector containing feasible set $\left\{\alpha_{k}\right\}$. It has been proved in [25] that F(·) is a convex function and G(·) is linear with respect to α. Hence SP is equivalent to the following quasi-convex problem:

(31) $$\begin{array}{c} \begin{array}{c} \;\;\;\min_{\begin{array}{c} \alpha \end{array}}\;\;\;\;\;\;\frac{\sigma^{2}}{G(\alpha )}\sum_{k=1}^{K}\frac{F(\alpha_{k})}{{\left|H\right|}_{k}^{2}\rho_{k}}\\ s.t.\;\;\;0<\alpha_{k}<{\hat{\alpha }}_{k}=\frac{\tilde{f}\left(\gamma_{k}\right)}{1+\tilde{f}\left(\gamma_{k}\right)} \end{array} \end{array}$$

where the upper bound ${\hat{\alpha }}_{k}$ is given according to constraint (22) in OP. Through utilizing standard convex optimization tool [26], the optimal solution to such quasi-convex problem can be obtained. The detailed solution procedure for such a convex problem can also be found in [25], which we would like to omit here for simplicity. Then with $\alpha^{*}=\left[\alpha_{1}^{*},\cdots ,\alpha_{K}^{*}\right]$ on hand, the optimal power assignment $\left\{P_{k}^{*}\right\}$ can also be achieved by plugging $\alpha^{*}$ into (28)–(26) inversely and consequently. Please confirm if it is (26)–(28) We then go back to (16) to check whether the EH and ID constraints are both satisfied with $\left\{P_{k}^{*}\right\}$. There might be two cases:

- **ID Dominated Case** happened when $P_{\mathrm{ID}}^{*}\geq P_{\mathrm{EH}}^{*}$, indicating RN needs more energy to achieve the target BER than that of EH. In such a case, $\left\{P_{k}^{*}\right\}$ can be directly applied as the final solution in current stage, while the RNs may harvest amount of energy that more than needed. We then can adaptively adjusting $\left\{\rho_{k}\right\}$ and $\left\{Q_{k}\right\}$ to re-balance the portion of energy assigned to EH and ID in the following step. The purpose is to leave less power for EH since the EH constraint is the weak one now and can be easily satisfied. While contrarily for ID function more power can be provided to help achieve its target. With the new setup of $\left\{\rho_{k}\right\}$ and $\left\{Q_{k}\right\}$ one can perform the power allocation iteratively to further reduce the total required transmit power.
- **EH Dominated Case** occurs, on the other hand, when $P_{\mathrm{ID}}^{*}<P_{\mathrm{EH}}^{*}$. In such situation, $\left\{P_{k}^{*}\right\}$ is no longer the feasible solution to satisfy the EH constraint since $\sum_{k=1}^{K}P_{k}^{*}<P_{\mathrm{EH}}^{*}$. In addition, it is impossible to get the exact power allocation profile of individual RN solely based on (13), who gives only the boundary of total transmit power. Nevertheless, we can proportionally increase $\left\{P_{k}^{*}\right\}$ to make it meet the EH requirement as ${\tilde{P}}_{k}^{*}=\eta P_{k}^{*}\;,\forall k$, where
  (32) $$\begin{array}{c} \eta =\frac{P_{\mathrm{EH}}^{*}}{\sum_{k=1}^{K}P_{k}^{*}}\; \end{array}$$
  is the proportional enlargement factor. By such operation, we keep the initial signal to interference ratio of each RN with ${\tilde{P}}_{k}^{*}$ remains the same as that with $P_{k}^{*}$. Apparently it guarantees the BER performance of RNs by scarifying more power than actually needs. Nevertheless, similar to that in **ID Dominated Case**, we can dynamically change $\left\{\rho_{k}\right\}$ and $\left\{Q_{k}\right\}$ to weaken the EH constraint, so that the total transmitter power might be further reduced.

### 3.2. Optimization of $\left\{Q_{k}\right\}$ and $\left\{\rho_{k}\right\}$ with Fixed $\left\{P_{k}\right\}$

Once $\left\{P_{k}^{*}\right\}$ obtained, we then could optimize $\left\{Q_{k}\right\}$ and $\left\{\rho_{k}\right\}$ in the next step. As aforementioned, to guarantee the convergence of MUD as well as to simplify the derivation, we initially set a relatively larger iteration number so that the assumption in (20) can hold. Such setup facilities the optimization of $\left\{P_{k}^{*}\right\}$ but leads to unnecessary power cost during EH phase. Hence with $\left\{P_{k}^{*}\right\}$ obtained in the previous step, we then track the actual iteration number that each RN needs to achieve its target SINR threshold based on (14). In other words, it is not required for the iterative MUD to be converged and the iteration would stop as long as the target SINR is reached. As f(·) is obtained beforehand, such recursive tracking process is of low complexity.

On the other hand, the optimal power splitting ratio can be linearly expressed as

(33) $$\begin{array}{c} \rho_{k}^{*}=1-\frac{\Pi_{k}^{*}}{\xi |H_{k}{|}^{2}N\sum_{k=1}^{K}P_{k}^{*}}\;,\forall k \end{array}$$

where $\Pi_{k}^{*}$ is determined by the actual iteration number $Q_{k}^{*}$ of each RN through tracking (14).

The updated $\left\{\rho_{k}^{*}\right\}$ and $\left\{Q_{k}^{*}\right\}$ are fed back to the previous step to once again optimize $\left\{P_{k}\right\}$. Note that the EH constraint should be updated by substituting the new parameters $\left\{\rho_{k}^{*}\right\}$ and $\left\{Q_{k}^{*}\right\}$ into (14), and $P_{\mathrm{EH}}^{*}$ should also be re-calculated accordingly.

The above procedure is cyclicly repeated until $P_{\mathrm{SN}}$ could not be reduced. Through such iterative optimization process, $\left\{P_{k}\right\}$, $\left\{\rho_{k}^{*}\right\}$ and $\left\{Q_{k}^{*}\right\}$ can be jointly optimized. The proposed sub-optimal scheme is concluded in Algorithm 1.

We then discuss the impact of $\left\{Q_{k}\right\}$ and $\left\{\rho_{k}\right\}$ on the power allocation solution in our proposed algorithm. As aforementioned, the minimum transmit power of SN is bounded by the larger one among $P_{\mathrm{EH}}^{*}$ and $P_{\mathrm{ID}}^{*}$, which are separately calculated considering their respective requirement, as shown in Section 3.1. The gap between them, suggests the unnecessary power cost of either one has to pay to fulfill the need of another. Intuitively speaking, the optimal power allocation should be the one to minimize such a gap. Note that both $P_{\mathrm{EH}}^{*}$ and $P_{\mathrm{ID}}^{*}$ are obtained with respect to certain pre-defined $\left\{Q_{k}\right\}$ and $\left\{\rho_{k}\right\}$. Hence although the EH and ID requirements, i.e., $\left\{\gamma_{k}\right\}$ and $\left\{E_{c}\right\}$ are determined, there is still chance to narrow the gap by varying the value of $\left\{\rho_{k}\right\}$ and $\left\{Q_{k}\right\}$, where the former may help to re-balance the portion of each RN’s power for the purpose of EH and ID, and the latter may help to renovate the EH requirement.
**Algorithm 1** The proposed sub-optimal Algorithm**Initialize:**1:Set I=0, $P_{\mathrm{SN}}^{0}=\infty$; $\rho_{k}=\rho ,\;Q_{k}=Q,\;\forall k\;;$2:Input f(·).**Iteration:**3:I←I+1;4:Compute $P_{\mathrm{EH}}$ according to (17);5:Compute $P_{\mathrm{ID}}=\sum_{k=1}^{K}P_{k}$:6: a) compute α in Problem (31), based on lagrange Multiplier Method and Karush-Kuhn-Tucker Condition;7: b) compute $\left\{\Gamma_{k}\right\}$ by inversely plugging α into (28) as: $\Gamma_{k}={\tilde{f}}^{-1}\left(\frac{\alpha_{k}}{1-\alpha_{k}}\right)\;;$8: c) comput $P_{k}$ by inversely plugging α and $\left\{\Gamma_{k}\right\}$ into (26);9:**if** $P_{\mathrm{EH}}\leq P_{\mathrm{ID}}$, **then**10:  **Output:** $\left\{P_{k}^{I}=P_{k}\right\}$; $P_{\mathrm{SN}}^{I}=P_{\mathrm{ID}}$;11:**else**12:  **Output:** $\left\{P_{k}^{I}=\eta P_{k}\right\}$ based on (32); $P_{\mathrm{SN}}^{I}=P_{\mathrm{EH}}$;13:**end if**14:**if** $P_{\mathrm{SN}}^{I}\leq P_{\mathrm{SN}}^{I-1}$ **then**15:  a) Compute $\left\{Q_{k}^{I}\right\}$ based on (14) with $\left\{\Gamma_{k}\right\}$ and $\left\{P_{k}^{I}\right\}$;16:  b) Compute $\left\{\rho_{k}^{I}\right\}$ based on (33) with $\left\{Q_{k}^{I}\right\}$ and $\left\{P_{k}^{I}\right\}$;17:  c) Go back to Line 3 with $\left\{Q_{k}^{I}\right\}$ and $\left\{\rho_{k}^{I}\right\}$ for a new iteration;18:**else**19:  **Break**;20:  **Output**: $\left\{P_{k}^{*}=P_{k}^{I}\right\}$, $\left\{Q_{k}^{*}=Q_{k}^{I}\right\}$ and $\left\{\rho_{k}^{*}=\rho_{k}^{I}\right\}$.21:**end if**

### 3.3. Feasibility of The Proposed Algorithm in Practical Applications

Finally, we further discuss the feasibility of the proposed system as well as that of the joint power allocation and splitting algorithm in practical applications. As aforementioned, the proposed system is implementable to the energy constrained scenario of 5 G, as it allows the RNs to wireless harvest the energy from RF signal through SWIPT, and also to enjoy the high spectral efficiency provided by OFDM-IDMA. Such benefit is obtained due to the fact that the proposed system is designed by combining OFDM-IDMA and SWIPT together. Hence the practical implementation of the proposed system relies on the feasibility of these two techniques. From the aspect of OFDM-IDMA, we found that the transceiver design is relatively mature nowadays and the field programmable gate array (FPGA) implementation for the iterative MUD with low complexity has already been realized [27]. While on the other hand for SWIPT, there are also emerging research works discussing the related circuitry implementation, where the positive views of realizing SWIPT in practical case has been reported [28,29]. In general, the feasibility of SWIPT relies mostly on the EH circuits and the energy-information division protocols at the receive nodes. The main concern of EH circuits is the imperfect property of the power conversion from RF signal to direct current electricity. Fortunately, we have already noticed that and properly adopted the non-linear model to approximate such conversion behavior [21]. That is to say, the non-linear property of power conversion has been taken into account when developing the jointly power allocation and splitting algorithm in our work. Hence the proposed algorithm turns out to match with the practical scenario. As for the energy-information division protocols, we employed the PS protocol in our work. It has been proved in [28] that PS is closer to the information theoretical optimum than other protocols. More importantly, with PS the functions of both EH and information decoding can be achieved by sharing single receive antenna. It facilitates the hardware design in the situation that there is strict constraint on the size of receive equipments.

## 4. Simulation Results and Discussions

In this section, simulation results are provided to evaluate the performance of the proposed scheme. We consider a OFDM-IDMA system with 512 subcarriers, where the whole bandwidth of 80 MHz is evenly divided. The repetition code of length 16 is employed to spread the RNs’ data over the subcarriers. The RNs are located to be 1 m away from the SN and the large scale fading is 30 dB with the path-loss exponent assumed to be 3. In addition, it also experiences the frequency selective multi-path Rayleigh fading with the number of fading paths L=6, from SN to each RN. The power spectrum density of noise is $\sigma^{2}=112$ dBm/Hz. For EH, the initial PS ratio and iteration number of all RNs are ρ=0.5 and Q=30, respectively. The EH circuit specifications of all RNs are a=0.14, b=15, and M=25.2 mW.

### 4.1. Performance Versus ID Requirements

**Figure 2:**
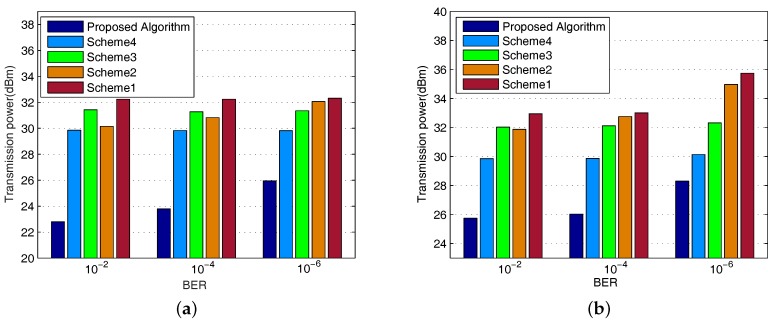
Transmit Power comparison with target BER equaling to $10^{-2}$, $10^{-4}$ and $10^{-6}$ ($E_{c}=5\;\mu$W, $\sigma^{2}=-112$ dBm). (**a**) K=8; (**b**) K=16.

We assume the RNs to have the same ID requirement and evaluate the performance of the proposed algorithm given the specific BER targets as shown in Figure 2. To compare, the performance of four refereed algorithms are also illustrated. For the simplicity of presentation, we termed them as follows:

**Scheme 1:** perform equal power allocation with fixed PS ratio and iteration number,
**Scheme 2:** perform adaptive power splitting with equal power allocation and fixed iteration number,
**Scheme 3:** perform adaptive power allocation with fixed PS ratio and iteration number,
**Scheme 4:** perform joint power allocation and power splitting with fixed iteration number.

We first evaluate the performance of the proposed scheme when K=8 and $E_{c}=5\;\mu$W, as illustrated in Figure 2a. It is observed that the proposed algorithm outperforms the other alternatives invariant of the BER targets. It is reasonable since the proposed algorithm considers all three factors that may have impact on the transmit power, i.e., power assignment, $\left\{\rho_{k}\right\}$ and $\left\{Q_{k}\right\}$. While the other schemes take none or only one to two parameters into consideration. It is also found that Scheme 2 performs better than Scheme 3 when BER requirement is low, while worse than the latter as the BER requirement increases. It reveals that power allocation owns higher impact on minimizing the transmitter power when ID constraint is stronger, while the PS ratio $\left\{\rho_{k}\right\}$ is more important when EH is the dominant constraint. In addition, the performance of Scheme 4 is superior than that of others except for the proposed algorithm. The main reason is owing to the fact that Scheme 4 do not consider $\left\{Q_{k}\right\}$. Henceforce the EH constraint might be over-estimated. However, it can be observed that the performance gap between the proposed algorithm and Scheme 4 turns out to be smaller when BER requirement rises from $10^{-2}$ to $10^{-6}$. Apparently, as BER target getting harder to achieve, the optimal power allocation problem falls into the **ID Dominated Case** as discussed in Section 3.1. To meet such a tough requirement, SN has to keep increasing the transmit power although the EH requirement has already been reached. In such scenario, the impact of $\left\{Q_{k}\right\}$ on the transmit power still remains but turns to be weaker. We further verify the performance of our proposed scheme by changing the number of RN K=16, which is shown in Figure 2b. The observation coincide with that of K=8 case. Nevertheless, we can find the system gets into **ID Dominated Case** at an earlier stage when the number of RNs get larger. That is mainly due to the even severer multi-user interference caused by containing more RNs, which makes the ID requirement to be harder to meet.

**Figure 3:**
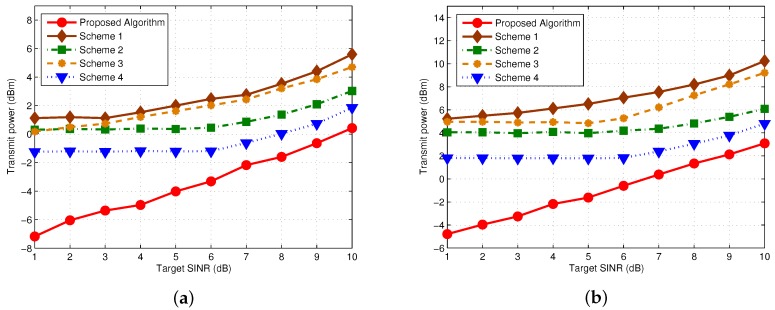
Transmit Power comparison versus target SINR ($E_{c}=5\;\mu$W, $\sigma^{2}=-112$ dBm). (**a**) K=8; (**b**) K=16.

To get a better insight into the impact of ID constraint on the performance, we further illustrated the resulted transmit power of various algorithms by varying the target SINR $\Gamma_{k}$ from 1–10 dB, as shown in Figure 3. The other setup parameters are the same as that in Figure 2. The observation here coincides with that in Figure 2. And we can find that as $\Gamma_{k}$ increases, the curves of proposed algorithm and Scheme 4 are getting closer and finally kept almost parallel with each other. Such observation shows that it still provide benefits to minimizing the total transmit power by involving $\left\{Q_{k}\right\}$ into the optimization process, even in **ID Dominated Case**.

### 4.2. Performance Versus EH Requirement

**Figure 4:**
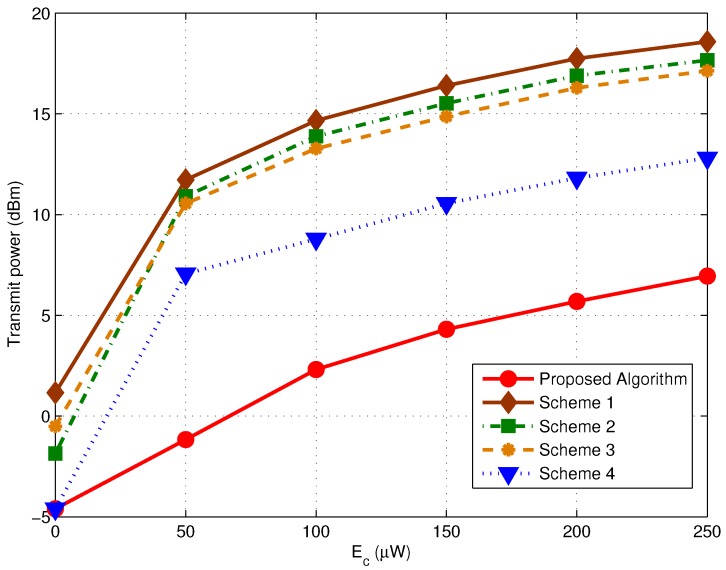
Transmit Power versus the harvest energy $E_{c}$ for the proposed algorithm ($\sigma^{2}=-112$ dBm and K=12, the BER target is $10^{-5}$ for each RN).

We then focus on the impact of EH Requirement on the performance of the proposed algorithm. Figure 4 depicts the minimum transmit power of various schemes versus $E_{c}$. We let K=12 with the BER target of each RN equals to $10^{-5}$. We can find the proposed algorithm always outperforms the other alternatives regardless of the $E_{c}$ value. In fact, the minimum required harvest energy of each RN is determined by $Q_{k}E_{c}$. It would provide more accurate expectation on the EH demands if we track the variation of $\left\{Q_{k}\right\}$ during the optimization process. It is of great importance in **EH Dominated Case**, where $E_{c}$ is relatively large and turns out to be the stronger constraints to the final transmit power.

**Figure 5:**
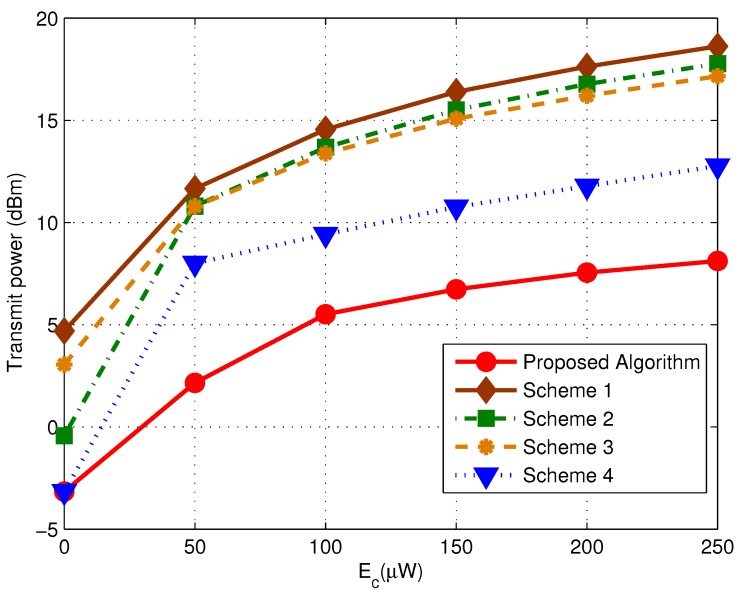
Transmit Power versus the harvest energy $E_{c}$ for the proposed algorithm ($\sigma^{2}=-112$ dBm and K=12, the RNs are evenly divided into 3 groups, with the corresponding BER target of each group $=10^{-2}$, $10^{-4}$ and $10^{-6}$).

We further verify our findings by set different BER targets among RNs, as illustrated in Figure 5. We let K=12 RNs to be evenly divided into 3 groups, with the BER target of each group equals to $10^{-2}$, $10^{-4}$ and $10^{-6}$, respectively. We can find the proposed algorithm is still advantageous over other schemes with any $E_{c}$ value. In addition, we observed that when $E_{c}$ is large, the total transmit power of each algorithm is smaller than the corresponding ones illustrated in Figure 4. It makes sense since the desired SINR of RNs with lower BER requirement has already been achieved, and less energy is needed.

**Figure 6:**
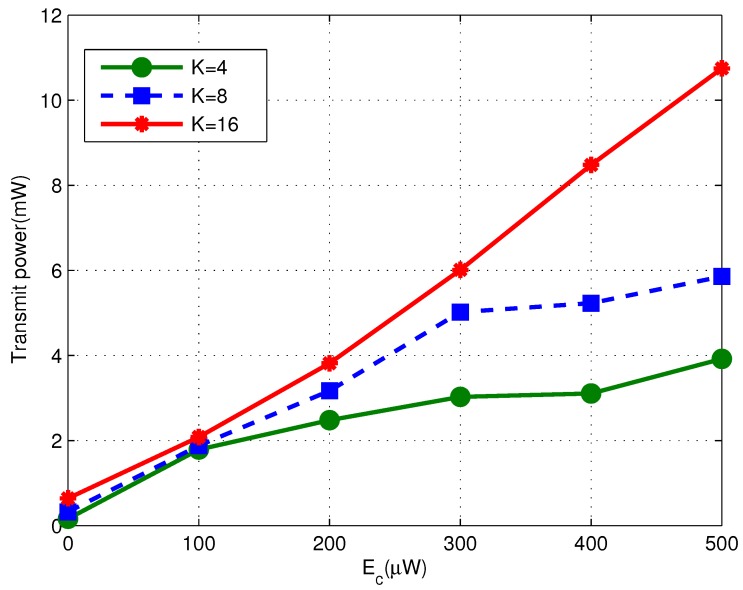
Transmit Power versus the harvest energy $E_{c}$ for the proposed algorithm ($\sigma^{2}=-112$ dBm and K=4,8,16).

In Figure 6 we plot the transmit power of the proposed algorithm with different number of RNs. Obviously, a larger number of RNs leads to higher transmit power. In addition, the slope of curves is getting smaller as $E_{c}$ increases, indicating that the power optimization problem shifts from the **ID Dominated Case** to the **EH Dominated Case**. Note that it would cause severer multi-user interference when more RNs are introduced into the system. Consequently the desire BER performance is harder to be achieved. Hence such transition process, along with the increase of $E_{c}$, is slower when the number of RNs is larger.

### 4.3. Performance of Block Error Rate

**Figure 7:**
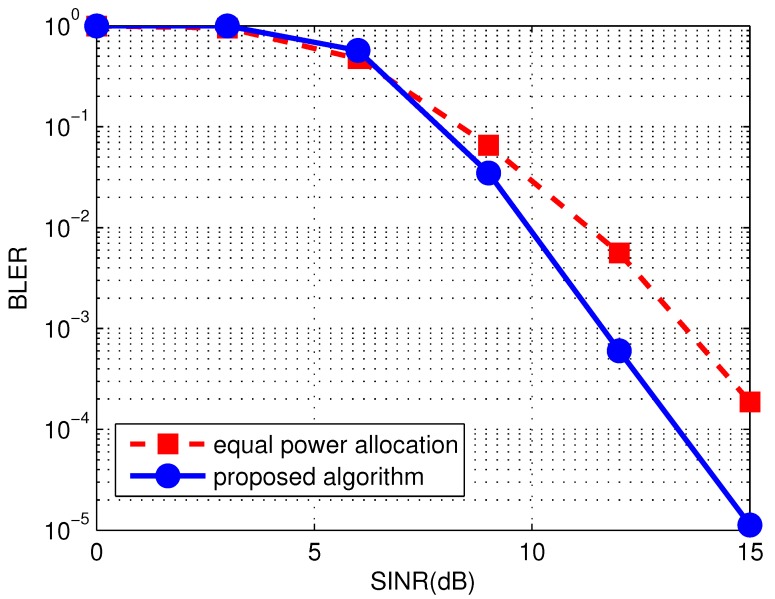
Block error rate (BLER) performance of SWIPT aided OFDM-IDMA system with the proposed power allocation algorithm and equal power allocation.The Cyclic Redundancy Check (CRC) sequence of length-16 is tailed at the end of each frame. K=16.

In Figure 7 we show the block error rate (BLER) of the proposed system, to evaluate the performance of the proposed algorithm from another perspective. To do so we first set the target BER to be $10^{-6}$ and get the power allocation profiles of different RNs with the proposed algorithm. Then we perform the simulations on BLER with such power allocation profiles by vary the SINR from 0–15 dB. To compare, we also illustrate the BLER performance of the system with equal power allocation. It can be observed from the figure that the BLER of the proposed system decreases as SINR increases, indicating the efficiency of the proposed system on cancelling the interference. Moreover, the system with our proposed algorithm outperforms the one with equal power allocation. And the performance gap becomes larger when SINR is higher. It reveals that the SWIPT aided OFDM-IDMA system with the proposed algorithm performs well especially in the high SINR regime.

### 4.4. Convergency of The Proposed Algorithm

**Figure 8:**
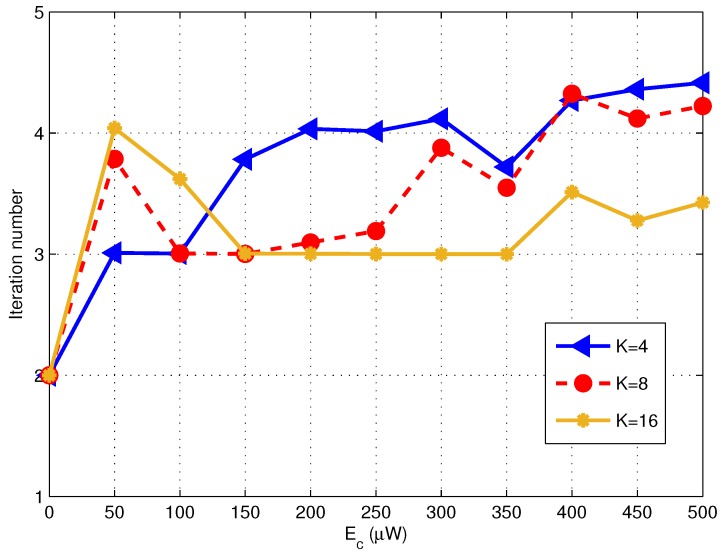
Iteration number needed for the proposed algorithm ($\sigma^{2}=-112$ dBm K=4,8,16 and BER targets $=10^{-6}$).

Finally, we examine the convergency of the proposed algorithm through tracking the iteration times during optimization process, as shown in Figure 8. The BER target is set to be $10^{-6}$ for all RNs, and we consider the cases when the number of RNs *K* equals to 4, 8, and 16, respectively. The simulations are performed for 1000 times of channel realizations and the results are obtained by taking the expectation of iteration times over all channel realizations. It can be found that the iteration time keeps below 5 for any case with arbitrary $E_{c}$ and *K*. It confoirms the convergency of the proposed algorithm, which is invariant of the number of RNs, $E_{c}$ and also $\Gamma_{k}$. And the complexity of the proposed algorithm is relatively low, with the final solution obtained with quite limited time of iterations.

## 5. Conclusions and Future Works

In this paper, we have investigated the joint power allocation and power splitting problem in SWIPT aided OFDM-IDMA for power limited networks. The purpose is to minimize the total transmit power, subject to the requirements of both EH and ID functions. Due to the features of iterative MUD employed in OFDM-IDMA, both EH and ID requirements are affected by the number of iterations. Hence, we have formulated the power minimization problem into the one to jointly optimize the power allocation, PS ratio as well as the number of iterations among all RNs. We then decomposed the original problem into two sub-problems where the EH and ID requirements are considered independently. A sub-optimal algorithm has been proposed to approach the solution of the original problem, by iteratively solving these two sub-problems. Simulation results have confirmed that the performance can be greatly improved through the proposed algorithm.

The future work lies in two aspects. First, in the proposed system perfect channel state information is assumed. Considering the practical scenario, further study on the robust design of the power allocation and splitting algorithm with respect to the channel estimation error needs to be addressed. Second, we will extend the proposed SWIPT aided OFDM-IDMA system into the multi-hop networks. In such a case, the information of SN will not be delivered to the RNs directly, but through the relaying of multiple intermediate nodes. The optimal path selection problem turns out to be an important issue to impact the energy efficiency of the whole system [30,31], which requires further investigation in the future.
